# State-level restorative justice policies and mental health disparities among girl dating violence survivors in the US: A lagged cross-sectional study

**DOI:** 10.1186/s12889-026-28413-x

**Published:** 2026-07-13

**Authors:** Laurel Sharpless, Anna Austin, Tiara C. Willie, Trace Kershaw, Beth Moracco, Luz Reyes

**Affiliations:** 1 Department of Health Behavior, University of North Carolina at Chapel Hill, 135 Dauer Drive, Chapel Hill, NC 27599 USA; 2 Department of Mental Health, Johns Hopkins University, 624 N. Broadway, Baltimore, MD 21205 USA; 3 Department of Social and Behavioral Sciences, Yale University, 60 College Street, New Haven, CT 06510 USA; 4 Department of Community Health Sciences, University of California, Los Angeles, 650 Charles E Young Drive South, Los Angeles, CA 90095 USA

**Keywords:** Adolescent dating violence, Restorative justice, Policy, Mental health, Health equity

## Abstract

**Introduction:**

Adolescent dating violence (ADV) is highly prevalent among girls in the United States (US) and increases risk for depression, suicide ideation, and suicide attempts. Restorative justice (RJ), a policy-based, survivor-centered approach to harm repair and accountability, may be a structural intervention to promote mental health among ADV girl survivors. However, little is known about whether and how state-level RJ policies are associated with mental health or whether these associations differ by ethnoracial identity. Therefore, this study examined (1) associations between adolescent-focused, state-level RJ policies and depressive symptoms, suicide ideation, and suicide attempts among ADV girl survivors and (2) differences across ethnoracial identities.

**Methods:**

Individual-level data were from the 2013–2019 waves of the Youth Risk Behavior Survey, a state-representative, cross-sectional survey conducted biennially among public and private schools with adolescents who are in 9th to 12th grade in the US (*N* = 30,330). State-level policy data were from a US RJ legislative database. Generalized estimating equations using log binomial regression were used to examine lagged associations between state RJ policy adoption and mental health outcomes among ADV girl survivors and test for effect measure modification by ethnoracial identity.

**Results:**

There were no associations between residing in a state with an RJ policy (vs. without) and depressive symptoms, suicide ideation, or suicide attempts among ADV girl survivors. The associations for depressive symptoms and suicide ideation did not vary by ethnoracial identity. However, the association between residing in a state with an RJ policy (vs. without) and attempting suicide varied by ethnoracial identity (b [95% CI] = 0.21 [0.05, 0.37], *p* = .008), such that Black ADV girl survivors had an *increased* risk, and white ADV girls survivors had a *decreased* risk of attempting suicide; the association did not vary for Hispanic ADV girl survivors.

**Conclusions:**

State-level RJ policies may reduce suicide attempts for white ADV girl survivors, but addressing structural gendered racism in policy implementation may be necessary to promote mental health equity for Black and Hispanic ADV girl survivors.

**Supplementary Information:**

The online version contains supplementary material available at https://doi.org/10.1186/s12889-026-28413-x.

## Introduction

Adolescent dating violence (ADV), defined as physical, sexual or psychological dating abuse, or stalking that can occur in-person or digitally [1], is highly prevalent among girls and is associated with adverse short- and long-term mental health outcomes [2, 3]. Psychological ADV is one of the most commonly reported forms of ADV and often co-occurs with physical or sexual ADV [4, 5]. National data estimate that 1 in 5 adolescent girls in the United States (US) experience physical and/or sexual ADV each year [6, 7]. Experiencing physical and/or sexual ADV can lead to detrimental mental health consequences. Specifically, adolescent girls who have experienced physical or sexual ADV are twice as likely to experience depression and six to nine times as likely to have experienced recent suicide ideation and/or suicide attempts than adolescent girls who have not experienced ADV [8], with temporal effects supported by longitudinal data [5, 9]. Additionally, prevalence estimates suggest that ADV may disproportionately impact Black and Hispanic adolescent girls than white adolescent girls [6, 10–13]. For example, nationally representative data suggest that 13.1% of Black girls, 9.2% of Hispanic girls, and 8.0% of white girls experience physical ADV [13]. Thus, it is important to identify effective depression and suicide prevention strategies for ADV girl survivors.

Restorative justice (RJ), a policy-based justice and accountability response to ADV, is a strategy that has the potential to improve mental health outcomes among ADV girl survivors [14–16]. RJ derives from a theory of justice rooted in indigenous practices that posits that humans thrive in relational contexts rather than contexts of social control [17]. Rather than focus on punishing people who have caused harm, RJ focuses on restitution of survivors through harm repair, resolution through decreased risk of harm recurrence, and reconciliation through emotional healing [17, 18]. RJ policies can create access to RJ practices specified in the policy [19]. These RJ practices can occur in a variety of forms, such as mediation, circles, or conferences [20, 21], but they typically involve bringing the ADV survivor and perpetrator, or person who caused harm, together in a safe and secure space for harm repair, which is mediated by a trained facilitator [22]. With this practice, the ADV survivor narrates their story of the harm they experienced from ADV, and the person who caused harm works to repair the harm and be rehabilitated [23]. Moreover, peers, family, and community members can be involved at various stages of the practice to promote education of ADV and healthy reintegration into society or schools [20–22]. In the context of ADV, RJ is considered an alternative or supplementary response to the juvenile justice system that offers a survivor-centered and solution-based structural approach to harm repair [14, 18].

While not specific to RJ policies, Komro and colleagues (2012) [16] developed a conceptual framework of the causal pathway through which policies can affect health outcomes, which we adapted to guide the current study’s objectives. The framework suggests that state RJ policies and their implementation may improve survivor’s mental health by improving their psychosocial environment. Specifically, RJ policies may alter their psychosocial environment by decreasing power dynamics between an ADV girl survivor and person who caused harm and increasing a survivor’s sense of safety, social support [24], social, interpersonal, and emotional skills, well-being [25] educational attainment [26], stress management [27], and healing [24], which, in turn, can decrease risk for depressive symptoms, suicide ideation, and suicide attempts [16, 28]. The framework also suggests that these pathways may vary by sociodemographic characteristics, such as ethnoracial identity [16].

### Intersectionality

Guided by the framework [16], state-level RJ policies may differentially impact mental health outcomes across different groups of girls, shaped by their intersecting social positions. An intersectionality theoretical framework posits that social identities, such as race, sex, socioeconomic status, sexual orientation, and immigration status, intersect at the individual level to reflect and embody interlocking systems of power and oppression, such as racism, sexism, classism, heterosexism, and xenophobia, at a structural level [29–31]. These systems are embedded in policies, cultural norms, practices, and beliefs, amplifying and reinforcing one another to produce mental health inequities across intersectional groups [32]. For Black and Hispanic ADV girl survivors, these intersecting forms of oppression may exacerbate the negative impacts of ADV on mental health [32], making it less likely that RJ praxis alone would buffer these adverse effects given that RJ praxis may be embedded in systems that have historically and modernly perpetuated structural gendered racism against Black and Hispanic girls. This is salient for Black and Hispanic ADV girl survivors since state RJ policies may specify RJ to be practiced in schools or juvenile justice systems, systems that have historically and modernly discriminated against girls with these intersecting identities [33, 34]. For example, Black and Hispanic girls face disproportionate representation in the juvenile justice system and greater exposure to punitive school discipline compared to white girls [26, 35, 36]. Without addressing the larger context that RJ is practiced in (i.e., context of structural gendered racism), these policies might not have the intended effect for Black and Hispanic girls. Therefore, it is important to understand whether state-level RJ policies potentially reduce, create, or exacerbate mental health disparities by ethnoracial identity among ADV girl survivors and potentially disrupt macro level factors that may contribute to disparities (i.e., racism and sexism).

### Current study

Although state adoption of RJ policies may serve as a promising tool to attenuate adverse mental health outcomes among violence survivors, limited research has examined this link, and almost no research has been conducted with ADV survivors [26, 37]. Prior research using nationally representative data found that adult women intimate partner violence (IPV) survivors had worse perceived mental health than women without IPV experiences, and this relationship was weakened in states with an RJ policy vs. states without [37]. Previous research also suggests that residing in a state with an RJ policy (vs. without) is associated with better perceived mental health for white but not Hispanic or Black adult women IPV survivors [37]. To our knowledge, no study has examined associations of state-level RJ policy adoption with ADV girl survivors’ mental health, and whether associations vary by intersecting identities.

This study used an intracategorical intersectional approach [38], examining differences in outcomes across one axis of identity *within* a group defined by another axis [39], to examine associations between adolescent-focused, state-level RJ policies and depressive symptoms, suicide ideation, and suicide attempts among ADV girl survivors and test for effect measure modification by ethnoracial group. We hypothesized that ADV girl survivors in states with an RJ policy would have a reduced risk of depressive symptoms, suicide ideation, and suicide attempts than survivors residing in states without an RJ policy. We further hypothesized that associations would be stronger for ADV girl survivors who identified as non-Hispanic white than non-Hispanic Black or Hispanic.

## Methods

### Study sample

Individual-level data were from waves 2013, 2015, 2017, and 2019 of the Youth Risk Behavior Survey (YRBS) and were restricted to girls who identified as female and reported experiencing at least 1 episode of physical or sexual ADV in the past 12 months. These waves were used given a change in 2013 in questions assessing ADV and potential COVID-19 pandemic influences for data collected after 2020. YRBS is a population-representative, cross-sectional survey conducted biennially among public and private schools with adolescents who are in 9th to 12th grade in the US [13, 40–42]. State-representative YRBS data are publicly available from the Centers for Disease Control and Prevention (CDC). States’ YRBS use a three-stage cluster sample design. The first stage catalogues counties, categorizes them by metropolitan status and ethnoracial composition, and samples counties with a probability proportional to overall school enrollment size for the county. The second stage employs a probability proportional to a school’s enrollment size selection method to select schools for inclusion. Within these schools, the third stage randomly selects classes that are complete and required, either for a specific subject or during a mandatory time slot (such as second period). Schools with high enrollment of minoritized ethnoracial groups have two classes randomly selected to oversample Black and Hispanic students. Every student in these randomly selected classes are then eligible to participate [43]. Following parental consent procedures and student assent, students complete a self-administered questionnaire with responses directly recorded on a computer-scannable booklet during a single class period. All study procedures were approved by the CDC Institutional Review Board [44]. State YRBS that included both physical and sexual ADV questions were included in the study sample. Four states, Colorado, Maine, Missouri, and North Dakota, lacked YRBS data that asked about both physical and sexual ADV and were excluded from analysis.

State-level policy data were from a US RJ legislative database [45] that was created by Drs. Shannon Sliva and Carolyn Lambert through a content analysis of criminal and juvenile codes referencing RJ via the Westlaw online research service [19]. Specifically, RJ was defined as “the practice of bringing together those who have a stake in a particular offense to repair the harms caused by crime and promote restoration and reconciliation, to the extent possible, between victim, offender, and community” [46]. When creating the database, Sliva and Lambert compared policies against this standard definition of RJ, and policies that either contained the phrase, “restorative justice” or practices that were understood to be RJ (i.e., victim-offender dialogue) were categorized as having an RJ policy in the database. The database is a compendium of state-level RJ policies in all 50 States and the District of Columbia and the year policies are enacted and potentially retracted. The current study explicitly examined state RJ policies from the database that are labeled as specific to adolescents, which is defined as youth under age 18 years. Access to the database was granted for the purposes of this study. Operationalization of state-level RJ policy variables accounted for a one-year lag to align with YRBS response frames which asked about past 12-month ADV, depressive symptoms, suicide ideation, and suicide attempts. For example, states that had an RJ policy adopted up to and including in 2012 were identified as having an RJ policy to align with the 2013 YRBS wave. Nine states and the District of Columbia lacked adolescent-focused RJ policy status data and were excluded from the sample. The present study received exemption by University of North Carolina at Chapel Hill Institutional Review Board given the use of de-identified, publicly available data.

### Measures

**Presence of individual-level depressive symptoms** was assessed using the question, “During the last 12 months, did you ever feel so sad and hopeless almost every day for two weeks or more in a row that you stopped doing some usual activities?” Response options were coded into a binary variable as 1 = yes and 0 = no.

**Individual-level suicide ideation** was assessed using the question, “During the past 12 months, did you ever seriously consider attempting suicide?” Response options were coded into a binary variable as 1 = yes and 0 = no.

**Individual-level suicide attempt** was assessed using the question, “During the past 12 months, how many times did you actually attempt suicide?” Response options ranged from 0 times to 6 or more times and were coded into a binary variable as 1 = 1 or more times and 0 = 0 times.

**Individual-level race and ethnicity** were assessed using the following questions: “Are you Hispanic or Latino?” with response options of yes or no, and “What is your race?” with response options of American Indian or Alaska Native, Asian, Black or African American, Native Hawaiian or Other Pacific Islander, and white, where more than one option could be chosen. Participants who respond, “yes” to the first question were categorized as Hispanic (including Hispanic, multiple race) regardless of the response to the second question. Participants who responded, “no” to the first question and identified only as Black or African American were coded as non-Hispanic Black, and participants who responded “no” to the first question and identified only as white were coded as non-Hispanic white [13]. Thus, these questions were was made into a categorical variable where 1 = non-Hispanic white, 2 = non-Hispanic Black, and 3 = Hispanic (including Hispanic, multiple race) and were dummy coded with white as the reference. Participants who did not identify as Hispanic, non-Hispanic Black or African American only, or non-Hispanic white only were removed from analysis given that if combined into a heterogeneous single category to yield sufficient sample size for analysis, results related to this category would be uninterpretable [47]. For numbers and percentages for all ethnoracial categories [48], see Table 1.

**Table 1:** Individual-level percentages of overall sample of physical and/or sexual ADV girl survivors

Characteristic	RJ* policy (*n* = 12778)		No RJ* policy (*n* = 17552)		Total (*N* = 30330)	
	No.	% (95% CI*)	No.	% (95% CI*)	No.	% (95% CI*)
Race and ethnicity						
Non-Hispanic Black	1133	9.0 (8.3–9.8)	2970	17.4 (16.5–18.2)	4103	13.8 (13.3–14.4)
Hispanic	2333	18.6 (17.4–19.8)	3696	21.6 (20.5–22.7)	6029	20.3 (19.5–21.2)
Non-Hispanic white	7736	61.7 (59.8–63.5)	8271	57.5 (54.9–60.1)	16,099	54.3 (53.3–55.4)
American Indian/Alaska Native	307	2.4 (2.1–2.8)	340	2.0 (1.6–2.3)	647	2.2 (1.9–2.4)
Asian	170	1.4 (1.1–1.6)	397	2.3 (2.1–2.6)	567	1.9 (1.7–2.1)
Native Hawaiian/Other Pacific Islander	76	0.6 (0.4–0.8)	168	1.0 (0.8–1.1)	244	0.8 (0.7–0.9)
Non-Hispanic multiple race	789	6.3 (5.9–6.7)	1157	6.8 (6.4–7.2)	1946	6.6 (6.3–6.9)
Type of ADV*						
Physical ADV* only	3802	29.8 (28.8–30.7)	5686	32.4 (31.6–33.2)	9488	31.3 (30.7–31.9)
Sexual ADV* only	5829	45.6 (44.6–46.6)	7116	40.5 (39.7–41.4)	12,945	42.7 (42.0–43.4)
Both physical and sexual ADV*	3147	24.6 (23.9–25.4)	4750	27.1 (26.3–27.8)	7897	26.0 (25.5–26.6)
Depressive symptoms						
Yes	8639	68.7 (67.8–69.6)	11,261	66.2 (65.5–66.9)	19,900	67.2 (66.7–67.8)
No	3941	31.3 (30.4–32.2)	5759	33.8 (33.1–34.5)	9700	32.8 (32.2–33.3)
Suicide ideation						
Yes	5464	48.1 (47.2–49.1)	7857	46.0 (45.3–46.8)	13,321	46.9 (46.3–47.5)
No	5892	51.9 (50.9–52.8)	9210	54.0 (53.2–54.7)	15,102	53.1 (52.5–53.7)
Suicide attempts						
Yes	3196	28.7 (27.9–29.5)	2151	30.4 (29.4–31.4)	5348	29.4 (28.7–30.0)
No	7940	71.3 (70.5–72.1)	4929	69.6 (68.6–70.6)	12,869	70.6 (70.0–71.3)

*ADV Adolescent dating violence, *RJ* Restorative justice, *CI* Confidence interval

Counts are unweighted; percentages are unweighted

**State-level RJ policy** was operationalized as a state-level statute that applies to adolescents and references the use of RJ [46]. A binary variable was generated to categorize states with and without an RJ policy adopted per state and year with 1 = present and 0 = absent.

**State-level control variables** included median household income, maximum Temporary Assistance for Needy Families (TANF) benefit for a family of 3, state minimum wage, refundable Earned Income Tax Credit, and Medicaid expansion [49–51]. Median household income and maximum TANF benefit for a family of 3 were treated as continuous variables. State minimum wage was made into a binary variable where states with a minimum wage that was higher than the federal minimum wage were coded as 1, and otherwise 0. Refundable Earned Income Tax Credit was coded to categorize states as 1 = present and 0 = absent. Medicaid expansion was made into a binary variable where states that adopted Medicaid expansion were coded as 1, and states that did not adopt Medicaid expansion were coded as 0. These were all time-varying variables, and ADV girl survivors were given a value corresponding to their state of residence at the time of the YRBS survey.

### Data analyses

Unweighted sociodemographic frequencies and percentages were calculated for physical and/or sexual ADV girl survivors in states with and without an adolescent-focused RJ policy and the overall sample. Unweighted generalized linear regression with a binomial distribution and log link was used to examine unadjusted associations between state RJ policies and mental health outcomes among ADV girl survivors. A conceptual diagram was developed, informed by existing empirical evidence and subject matter expertise [52], to identify variables that were likely to operate in the associations between state adoption of an RJ policy and the mental health outcomes among ADV girl survivors (Supplemental Fig. 1) [53]. For example, prior research suggests that broader state policy contexts and economic conditions shape patterned adoption of policies across states [54, 55]. Additionally, a growing area of research suggests that state-level policies and economic conditions, including Medicaid expansion, refundable Earned Income Tax Credit, minimum wage, TANF, and household income, are associated with mental health among youth or adult populations [56–59]. Based on the conceptual diagram, models were adjusted for ethnoracial group and measures of the underlying state policy context (maximum TANF benefit for a family of 3, state minimum wage, refundable Earned Income Tax Credit, and Medicaid expansion) and state economic conditions (median household income) to control for potential confounding bias [60]. Additional individual-level sociodemographic characteristics were not adjusted for in models since it was unlikely that these characteristics would be associated with likelihood of a state adopting an RJ policy given that they would not capture the overall state policy context. All models used a generalized estimating equations approach with an exchangeable working correlation matrix and robust standard errors to account for clustering of adolescents within state, and included year as a fixed effect. Additionally, all analyses included stratification and clustering (primary sampling unit) variables to account for the complex sampling design used in YRBS. Ethnoracial group was assessed as a potential effect modifier by adding RJ policy x ethnoracial group interaction terms to adjusted models for each ethnoracial group except for the referent group (non-Hispanic white) to provide intracategorical comparisons. Consistent with the approach indicated by Aiken & West, significant interactions were probed using post-hoc analysis to test simple slopes [61]. Model coefficients, denoted as betas, risk ratios, and adjusted risk ratios, and the width and location of corresponding 95% confidence intervals were used to interpret results of analyses. *P*-values were not over-relied on in accordance with guidance from the American Statistical Association [62]. All analyses were conducted using SAS statistical software [63].

YRBS offers weights that are designed to produce population-representative estimates of students in grades 9–12 in US High Schools by adjusting for student sex, grade, and race and ethnicity [43]. The current analyses are not weighted because appropriate weights for the restricted sample of ADV girl survivors used in this study were not available. Ethnoracial group is the only weight-based variable in this study’s analyses, so subsample-specific weights of ethnoracial identity were needed to adjust for overrepresentation and accurately reflect ADV girl survivors in the US population but were unavailable. Therefore, the present study assumed that within each level of ethnoracial group of the sample of ADV girl survivors, the sample contains properties of a simple random sample, and thus, produces unbiased estimates [64, 65].

## Results

**Fig. 1:**
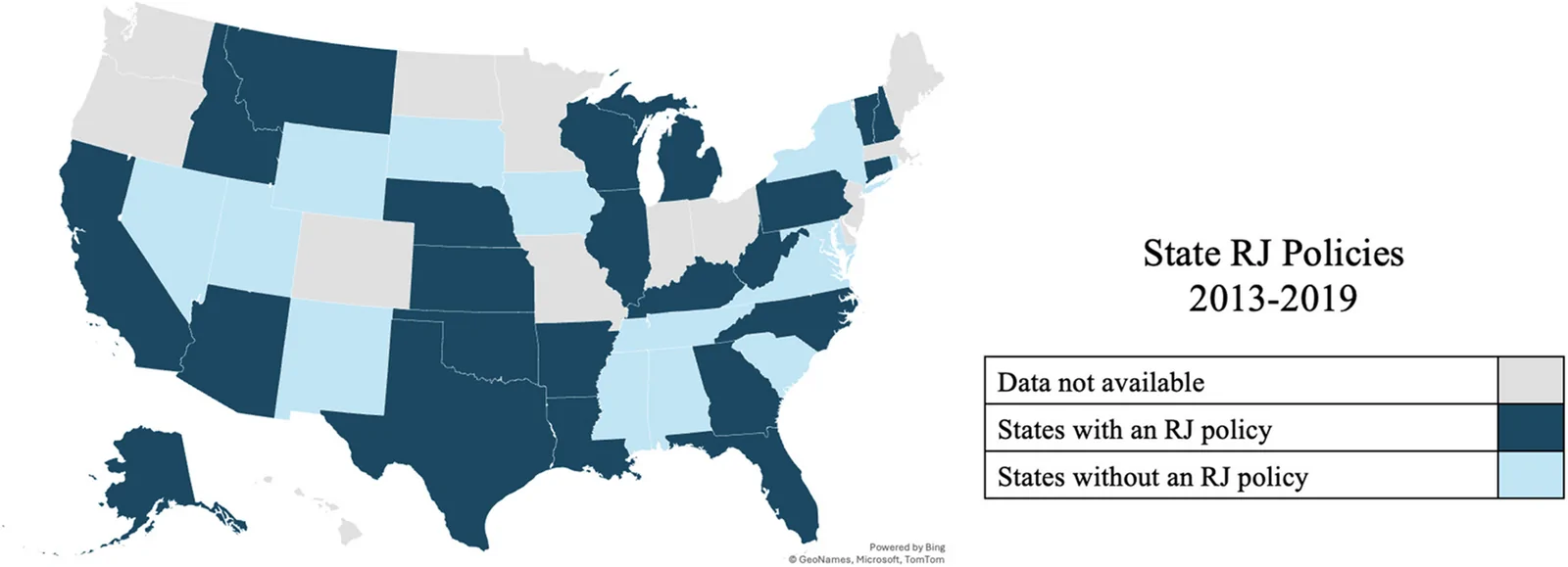
Map of distribution of states with and without RJ policies. *RJ = restorative justice. Arkansas, Connecticut, Georgia, Kentucky, Michigan and West Virginia adopted an RJ policy during the study period

Figure 1 displays the state-level distribution of adolescent-focused RJ policy status in the US during the 2013–2019 study period. 23 states had an RJ policy in place at any point throughout 2013–2019, while 14 states did not. Of the 23 states that had an RJ policy in place, Arkansas, Connecticut, Georgia, Kentucky, Michigan and West Virginia adopted an RJ policy during the study period.

### State-level potential confounders

Supplemental Table 1 displays the distribution of potential state-level confounders across state-years with and without an RJ policy adopted. Among the 60 state-years *with* an RJ policy, the average maximum TANF benefit for a family of 3 was $954.20 compared to $957.10 in state-years without an RJ policy. The average median household income was $55,081.00 in state-years *with* an RJ policy compared to $54,000.50 in state-years without. Moreover, in state-years *with* an RJ policy, 27 state-years had a minimum wage that was higher than the federal minimum wage (24.8%), 21 state-years had a state refundable Earned Income Tax Credit rate (19.3%), and 26 state-years had expanded Medicaid (23.9%). Whereas, in state-years without an RJ policy, 21 state-years had a minimum wage that was higher than the federal minimum wage (19.3%), 21 state-years had a state refundable Earned Income Tax Credit rate (19.3%), and 19 state-years had expanded Medicaid (17.4%).

### Sociodemographic characteristics

Table 1 shows unweighted frequencies and percentages of sociodemographic characteristics of the overall sample of 30,330 physical and/or sexual ADV girl survivors who participated in YRBS during the study period. Of these girls, 12,778 were in state-years *with* an RJ policy, while 17,552 were in state-years without an RJ policy. Among ADV girl survivors in state-years *with* an RJ policy, 61.7% self-identified as non-Hispanic white, 18.6% identified as Hispanic, and 9% identified as non-Hispanic Black. Among ADV girl survivors in state-years without an RJ policy, 57.5% identified as non-Hispanic white, 21.6% identified as Hispanic, and 17.4% identified as non-Hispanic Black. In state-years *with* an RJ policy, 29.8% of girls experienced physical ADV only, 45.6% of girls experienced sexual ADV only, and 24.6% experienced both physical and sexual ADV. In state-years without an RJ policy, 32.4% of girls experienced physical ADV only, 40.5% of girls experienced sexual ADV only, and 27.1% experienced both physical and sexual ADV. Further, 68.7% of ADV girl survivors in state-years *with* an RJ policy reported experiencing depressive symptoms, 48.1% had suicide ideation, and 28.7% attempted suicide. Whereas 66.2% of ADV girl survivors in state-years without an RJ policy experienced depressive symptoms, 46.0% had suicide ideation, and 30.4% attempted suicide.

### Multivariable associations between state RJ policies and depressive symptoms

Unweighted unadjusted and adjusted associations were examined between state RJ polices and depressive symptoms (Table 2). Contrary to expectations, unadjusted results showed that ADV girl survivors who resided in states *with* an RJ policy had 1.04 times the risk of depressive symptoms compared to ADV girl survivors in states without an RJ policy (95% CI [1.02, 1.06], *p* <.001). After controlling for covariates, this association was attenuated and was no longer statistically significant (ARR [95% CI] = 0.98 [0.96, 1.00], *p* =.065).

**Table 2:** Associations between state RJ policies and depressive symptoms among ADV girl survivors

	Depressive symptoms					
	Unadjusted			Adjusted		
	RR	95% CI*	*p*-value	ARR*	95% CI*	*p*-value
RJ* policy						
Yes	1.04	(1.02, 1.06)	< 0.001	0.98	(0.96, 1.00)	0.065
No	Ref					
Race and ethnicity						
Non-Hispanic Black	0.89	(0.87, 0.92)	< 0.001	0.91	(0.89, 0.94)	< 0.001
Hispanic	1.08	(1.06, 1.10)	< 0.001	1.06	(1.04, 1.08)	< 0.001
Non-Hispanic white	Ref					
Median household income	1.00	(1.00, 1.00)	0.312	1.00	(1.00, 1.00)	0.832
Maximum TANF* benefit for family of 3	1.00	(0.99, 1.00)	0.170	1.00	(1.00, 1.00)	0.179
State minimum wage						
Higher than federal minimum wage	1.05	(1.03, 1.07)	< 0.001	1.01	(0.99, 1.03)	0.265
Lower than federal minimum wage	Ref					
State refundable EITC* rate						
Present	0.95	(0.94, 0.97)	< 0.001	0.96	(0.94, 0.98)	< 0.001
Absent	Ref					
Medicaid expansion						
Yes	1.06	(1.04, 1.08)	< 0.001	1.03	(1.01, 1.06)	0.019
No	Ref					

*RJ Restorative justice, *TANF* Temporary assistance for needy families, *EITC* Earned income tax credit, *ARR* Adjusted risk ratio, *CI* Confidence interval. Analyses are unweighted

Contrary to expectations, the RJ policy x Hispanic and RJ policy x Black interaction terms were not statistically significant in models for depressive symptoms; see Supplemental Table 2.

### Multivariable associations between state RJ policies and suicide ideation

Unweighted unadjusted and adjusted associations were examined between state RJ polices and suicide ideation (Table 3). Contrary to expectations, unadjusted results showed that ADV girl survivors who resided in states *with* an RJ policy had 1.05 times the risk of suicide ideation compared to ADV girl survivors in states without an RJ policy (95% CI [1.02, 1.08], *p* <.001). After controlling for covariates, this association was attenuated and was no longer statistically significant (ARR [95% CI] = 0.99 [0.96, 1.02, *p* =.537).

**Table 3:** Associations between state RJ policies and suicide ideation among ADV girl survivors

	Suicide Ideation					
	Unadjusted			Adjusted		
	RR	95% CI*	*p*-value	ARR*	95% CI*	*p*-value
RJ* policy						
Yes	1.05	(1.02, 1.08)	< 0.001	0.99	(0.96, 1.02)	0.537
No	Ref					
Race and ethnicity						
Non-Hispanic Black	0.84	(0.80, 0.87)	< 0.001	0.84	(0.80, 0.87)	< 0.001
Hispanic	1.02	(0.99, 1.06)	0.138	0.99	(0.96, 1.03)	0.620
Non-Hispanic white	Ref					
Median household income	1.00	(1.00, 1.00)	0.066	1.00	(1.00, 1.00)	0.002
Maximum TANF* benefit for family of 3	1.00	(0.99, 1.00)	0.344	1.00	(1.00, 1.00)	< 0.001
State minimum wage						
Higher than federal minimum wage	1.02	(0.99, 1.05)	0.164	1.01	(0.97, 1.04)	0.613
Lower than federal minimum wage	Ref					
State refundable EITC* rate						
Present	0.94	(0.92, 0.97)	< 0.001	0.95	(0.91, 0.98)	0.005
Absent	Ref					
Medicaid expansion						
Yes	1.08	(1.05, 1.11)	< 0.001	1.02	(0.98, 1.07)	0.303
No	Ref					

*RJ Restorative justice, *TANF* Temporary assistance for needy families, *EITC* Earned income tax credit, *ARR* Adjusted risk ratio, *CI* Confidence interval. Analyses are unweighted

Contrary to expectations, the RJ policy x Hispanic and RJ policy x Black interaction terms were not statistically significant in models for suicide ideation; see Supplemental Table 2.

### Multivariable associations between state RJ policies and suicide attempt

Unweighted unadjusted and adjusted associations were examined between state RJ polices and suicide attempts (Table 4). Unadjusted results showed that ADV girl survivors who resided in states *with* an RJ policy had 0.96 times the risk of attempting suicide compared to ADV girl survivors in states without an RJ policy (95% CI [0.91, 1.01], *p* =.093). Contrary to expectations, ADV girl survivors who resided in a state with an RJ policy had 0.98 times the risk of attempting suicide compared to ADV girl survivors in states without an RJ policy after controlling for covariates (95% CI [0.93, 1.04], *p* =.559).

**Table 4:** Associations between state RJ policies and attempting suicide among ADV girl survivors

	Suicide Attempts					
	Unadjusted			Adjusted		
	RR	95% CI*	*p*-value	ARR*	95% CI*	*p*-value
RJ* policy						
Yes	0.96	(0.91, 1.01)	0.093	0.98	(0.93, 1.04)	0.559
No	Ref					
Race and ethnicity						
Non-Hispanic Black	1.09	(1.01, 1.18)	0.024	1.19	(1.10, 1.28)	< 0.001
Hispanic	1.30	(1.23, 1.37)	< 0.001	1.36	(1.28, 1.44)	< 0.001
Non-Hispanic white	Ref					
Median household income	1.00	(1.00, 1.00)	0.003	1.00	(1.00, 1.00)	0.004
Maximum TANF* benefit for family of 3	1.00	(0.99, 1.00)	< 0.001	1.00	(0.99, 1.00)	0.600
State minimum wage						
Higher than federal minimum wage	0.98	(0.93, 1.03)	0.356	0.93	(0.88, 0.99)	0.022
Lower than federal minimum wage	Ref					
State refundable EITC* rate						
Present	0.94	(0.89, 0.99)	0.015	0.94	(0.88, 1.00)	0.041
Absent	Ref					
Medicaid expansion						
Yes	1.00	(0.95, 1.05)	0.895	1.03	(0.96, 1.10)	0.423
No	Ref					

*RJ Restorative justice, *TANF* Temporary assistance for needy families, *EITC* Earned income tax credit, *ARR* Adjusted risk ratio, *CI* Confidence interval. Analyses are unweighted

**Fig. 2:**
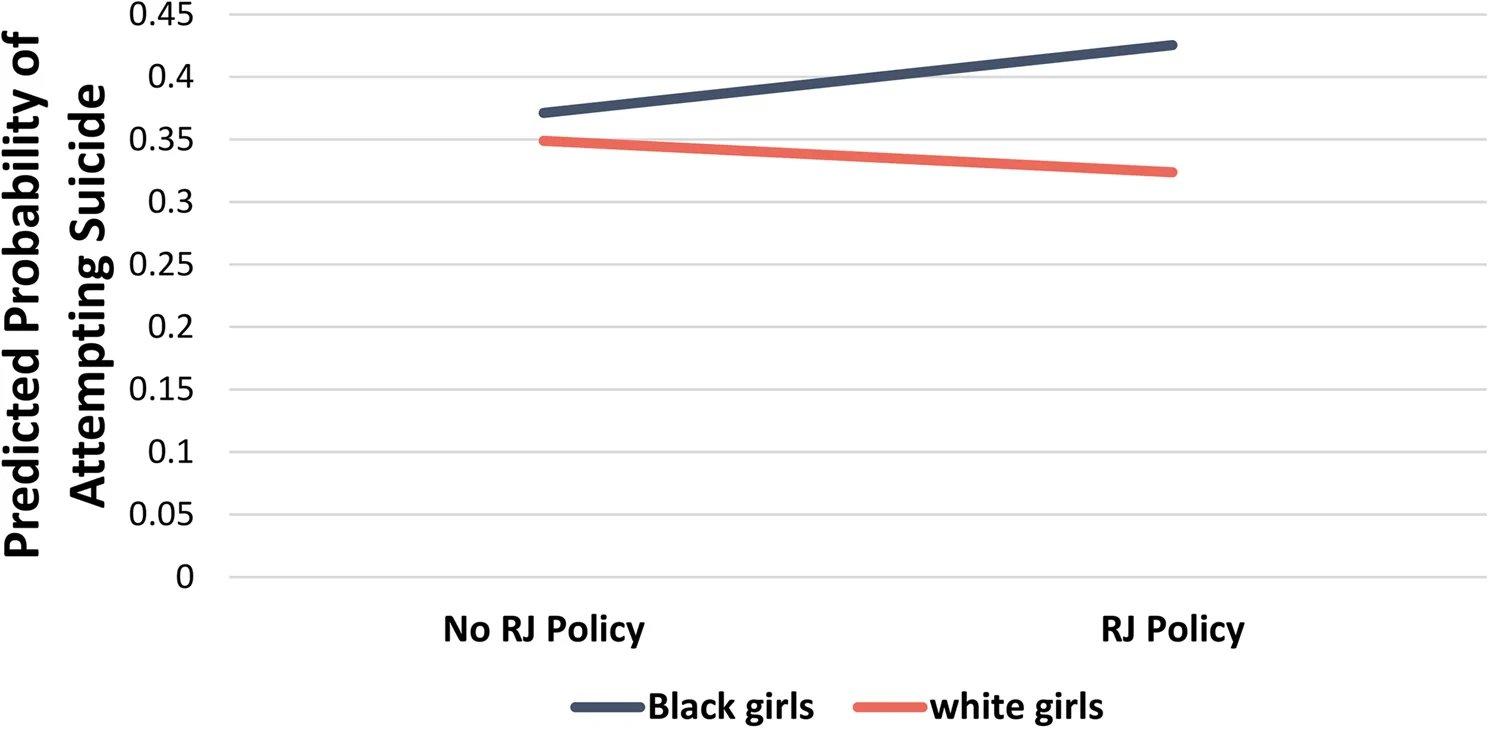
Interaction between RJ policy adoption and non-Hispanic Black identity on suicide attempts

In models predicting suicide attempts, the RJ policy x Black interaction was statistically significant in the model predicting risk of suicide attempt (b [95% CI] = 0.21 [0.05, 0.37], *p* =.008). Post-hoc probing of the interaction term showed that Black ADV girl survivors residing in states *with* an RJ policy had 1.15 times the risk of attempting suicide than Black ADV girl survivors in states without an RJ policy (95% CI [0.99, 1.31], *p* =.051). In contrast, white ADV girl survivors residing in states with an RJ policy had 0.93 times the risk of attempting suicide than white ADV girl survivors in states without an RJ policy (95% CI [0.86, 1.00], *p* =.054); see Fig. 2. Contrary to expectations, the RJ policy x Hispanic interaction term was not statistically significant for suicide attempts; see Supplemental Table 2.

We conducted a sensitivity analysis using the number of suicide attempts in adjusted models given that this is a different way to look at suicide attempts that retains more variability in this outcome and captures survivors who attempt suicide at least once vs. have repeated attempts. The variable response options ranged from 0 attempts to 6 or more attempts. ADV girl survivors who resided in a state with an RJ policy had 0.97 times the risk of attempting suicide compared to ADV girl survivors in states without an RJ policy after controlling for covariates (95% CI [0.90, 1.03], *p* =.307), and the RJ policy x Black interaction term was statistically significant in the model predicting risk of suicide attempts (b [95% CI] = 0.23 [0.05, 0.42], *p* =.015). Post-hoc probing of the interaction term showed that Black ADV girl survivors residing in states *with* an RJ policy had 1.13 times the risk of attempting suicide than Black ADV girl survivors in states without an RJ policy (95% CI [0.96, 1.34], *p* =.139). In contrast, white ADV girl survivors residing in states with an RJ policy had 0.90 times the risk of attempting suicide than white ADV girl survivors in states without an RJ policy (95% CI [0.82, 0.98], *p* =.018); The RJ policy x Hispanic interaction term was not statistically significant for risk of suicide attempts. Considering the magnitude and direction of risk and the width and location of the 95% confidence intervals, these results are consistent with above findings.

We also conducted a sensitivity analysis that included a measure of state political context as an additional time-varying covariate in the main adjusted models. This variable was coded in the original source as the state governor being 1 = Democrat and 0 = Republican [50]. Here, ADV girl survivors who resided in states *with* an RJ policy had 0.98 times the risk of depressive symptoms (95% CI [0.96, 1.01], *p* =.132), 0.99 times the risk of suicide ideation (95% CI [0.96, 1.03], *p* =.624), and 0.98 times the risk of attempting suicide compared to ADV girl survivors in states without an RJ policy (95% CI [0.93, 1.04], *p* =.542). These results are highly consistent with the primary findings.

## Discussion

To our knowledge, this is one of the first studies to examine associations between state-level RJ policies and mental health outcomes among ADV girl survivors. Contrary to expectations, we found no main associations between residing in a state with an RJ policy (vs. without) and depressive symptoms or suicide ideation, and these associations did not vary by ethnoracial group. Consistent with expectations, state-level RJ policies were associated with lowered risk of suicide attempt for white girls but, unexpectedly, greater risk for Black girls. However, unexpectedly, this association did not vary for Hispanic ADV girl survivors. Our findings for ADV girl survivors’ mental health are mixed and reveal more complexity compared to previous research on state RJ policies’ association with adult women IPV survivors’ mental health [37].

The protective association between residing in states with an RJ policy (vs. without) and attempting suicide among white ADV girl survivors is consistent with previous research conducted among adult women IPV survivors. In particular, Sharpless et al. [37] found that residing in a state with an RJ policy (vs. without) is associated with better perceived mental health for white adult women IPV survivors. However, the potentially harmful association between state-level RJ policies and attempting suicide among Black ADV girl survivors contrasts from prior findings, as Sharpless et al. [37] found no association between state RJ policies and perceived mental health among Black adult women IPV survivors. Our findings are also consistent with mixed evidence from evaluations of district-level RJ policies implemented within schools, which suggests that policies do not inherently reduce mental health disparities among middle and high school students, and that these associations may depend on key implementation features of the RJ policy [26]. There are several potential explanations for the divergent findings found in our study for white and Black ADV girl survivors, including variability in RJ program implementation, differences in facilitators’ training and practices, and broader community contexts that may shape how RJ practices are implemented and practiced [25]. One potential explanation may be due to the inequitable implementation of RJ praxis within schools and juvenile justice systems through RJ policies, shaped by structural gendered racism [66]. Ideally, RJ praxis should be effective for all ADV girl survivors, but those facilitating RJ practices may discriminate against Black ADV girl survivors based on their intersecting identities, resulting in harm for Black survivors [33, 34]. For example, Black girls can be stereotyped as aggressive, unmanageable, and hypersexual, which can lead to discriminatory subjective interpretation of Black girls’ behavior that reinforces punitive rather than restorative responses when addressing ADV [67]. As mentioned in the policy-to-mental health framework that guided this study [16], RJ practices can facilitate protective mechanisms that can improve a survivor’s psychosocial environment [24–27] and potentially promote mental health [16, 28], but these protective mechanisms may not be equitably accessible for Black ADV girl survivors if these stereotypes are accepted across settings. Conversely, white ADV girl survivors may undergo survivor-centered RJ practices in these settings where they are heard and supported [23] rather than discriminated against, allowing them to access intended RJ protective mechanisms, which can promote their mental health. When policies exist but fail to provide meaningful support to marginalized groups, they may not only be ineffective, potentially explaining null results for Hispanic ADV girl survivors, but could also contribute to further harm by reinforcing feelings of oppression [68]. Future research should examine how state-level RJ policies are implemented in practice, including qualitative work to understand how survivors experience these processes and how implementation varies across settings. This work can help ensure that RJ policies are contextually responsive and address structural gendered racism that may prevent Black and Hispanic ADV girl survivors from experiencing a positive mental health impact from RJ interventions or contribute to harm.

Given the preliminary evidence that suggests that state RJ policies may increase risk of attempting suicide among Black ADV girl survivors and be ineffective for Hispanic ADV girl survivors, transformative justice, a longstanding practice within Black and Hispanic communities, may offer a more effective approach to justice and accountability for these intersectional groups [69]. Transformative justice not only shares RJ’s core tenants and practices of harm repair but goes further by addressing the survivor’s broader social structural context. While RJ alone may not sufficiently address the structural gendered racism that Black and Hispanic ADV girl survivors may face, transformative justice embeds this awareness into its practices. Unlike RJ, which is often implemented within institutions, transformative justice is a distinctly community-led approach that reimagines justice without reliance on racist, sexist structures and seeks to transform the community conditions that contribute to violence [23, 69–71]. Emerging qualitative research among adult Black women IPV survivors highlights their preference for justice responses that align with transformative justice principles [72], which may be relevant for Black and Hispanic ADV girl survivors since both groups navigate structural gendered racism and may share mistrust in formal help-seeking systems. For example, extant research has shown that Black and Hispanic ADV girl survivors prefer informal help-seeking services for ADV, such as from parents or friends, rather than from formal services due to fear and mistrust [73]. Future research should extend this work to examine preferences for justice and accountability for ADV among Black and Hispanic ADV girl survivors, offering critical insights to inform a tailored justice approach for these groups that may align with transformative justice and potentially support their mental health. Since schools may be Black and Hispanic ADV girl survivors’ primary community, innovative approaches to integrating justice frameworks in these spaces may be needed. For example, school-community partnerships may be a strategy to bring transformative justice praxis to Black and Hispanic ADV girl survivors [74]. Future research in this area is warranted.

It is important to note that there were no significant associations found between residing in states with an RJ policy (vs. without) and depressive symptoms or suicide ideation, potential precursors to suicide attempts, for any ethnoracial groups. Some evidence suggests heterogeneity in mental health outcomes across ethnoracial groups [75–77]. For example, national YRBS data indicate that white adolescents are more likely to report suicide ideation compared to Black adolescents, yet this pattern reverses for suicide attempts, with Black adolescents being more likely to attempt suicide than white adolescents [78], and this pattern aligns with our data. However, it is unclear why an association was observed for only the most severe and least prevalent outcome, suicide attempts, among Black and white ADV girl survivors. Future research should replicate these findings and examine these mental health disparities in greater depth.

### Limitations

The present study has several limitations that should be considered. This is a pooled cross-sectional study, so we cannot infer causality with our study findings. Future research could examine YRBS surveys longitudinally to establish temporality between state RJ policies and mental health outcomes across diverse ADV girl survivors. Longitudinal analysis for the current study would have required additional years of YRBS data that were not yet collected at the time of analysis. This study used an existing RJ legislative database that contained RJ policies with varying texts and specifications. Future research can explore nuances of these policies in terms of their criteria on mental health outcomes for survivors. Although our study controlled for potential confounders of the state policy context and state economic conditions, this study did not adjust for additional policies and may not have controlled for all potential state-level confounders. Our sample is limited to ADV girl survivors who are enrolled in school, which excludes those who may have dropped out or are incarcerated. These groups may have unique experiences and should be a focus of future research. Moreover, the sample is limited to girls who reported ADV. Given the nature of ADV, there may have been girls who did not disclose ADV on YRBS, who were then excluded from the study sample, which could lead to selection bias. The severity of physical and/or sexual ADV experienced in this sample is not reflected in this study. Future research could examine how ADV severity may impact associations. Additionally, the YRBS surveys did not ask about psychological ADV or stalking in the waves used, which may miss ADV girl survivors and bias our results. Future work could use the 2023 YRBS wave given that psychological ADV has been added to this survey in some states, and future nationally representative surveys should measure these additional forms of ADV to more comprehensively assess the full range of ADV experiences using nationally representative data. The YRBS surveys used did not include questions about gender identity, preventing us from differentiating the experiences of cisgender ADV girl survivors from those who are transgender or non-binary. While our study emphasizes racism and sexism, future research should explore other intersecting systems of power and oppression. We used an intracategorical intersectional approach to our analyses, which may not capture the full complexities of intersectional experiences among ADV girl survivors. Survivors outside the three ethnoracial groups analyzed were also excluded due to insufficient sample size. Future studies should strive for a more diverse and representative sample of ADV girl survivors. Lastly, our findings are not representative of all states in the US and may not generalize to all ADV girl survivors in the US or to ADV girl survivors in countries outside of the US. Future work can produce population-representative estimates among ADV girl survivors in the US and other contexts.

## Conclusions

Our study findings provide new knowledge about the relationships between state-level RJ policies and mental health outcomes among ADV girl survivors, and how associations differ by ethnoracial identity. State-level RJ policies may be a promising scalable tool to reduce suicide attempts only for white ADV girl survivors. Ensuring that state RJ policies are not perpetuating structural gendered racism may ensure these policies do not cause harm for Black ADV girl survivors or are ineffective for Hispanic ADV girl survivors.

## Supplementary Information

Supplementary Material 1

## Data Availability

The data used for this study are publicly available from the Centers for Disease Control and Prevention Youth Risk Behavior Survey website and Graduate School of Social Work Restorative Justice Laws website.

## Abbreviations

ADV: Adolescent dating Violence
RJ: Restorative Justice
US: United States
YRBS: Youth Risk Behavior Survey
IPV: Intimate partner violence
CDC: Centers for Disease Control and Prevention
TANF: Temporary Assistance for Needy Families
