# Supplementary material for: State-level restorative justice policies and mental health disparities among girl dating violence survivors in the US: A lagged cross-sectional study
Source: BMC Public Health. 2026 Jul 13;26:2846. doi: 10.1186/s12889-026-28413-x (PMC13644015; doi:10.1186/s12889-026-28413-x)
Supplement: Supplementary file 1 [file 12889_2026_28413_MOESM1_ESM.docx]

###### Supplemental Figure 1 Conceptual diagram of potential confounders and demographic covariates

*ADV = adolescent dating violence; RJ = restorative justice; EITC = earned income tax credit; TANF = temporary assistance for needy families

##### Supplemental Table 1 State-level percentages of states that asked about physical and sexual ADV in YRBS

| Characteristic | RJ Policy  (n=60) | No RJ Policy  (n=49) | Total  (N=109) |
| --- | --- | --- | --- |
|  | N (%) | N (%) | N (%) |
| Maximum TANF benefit for family of 3 | 954.2 (795, 904, 1096) | 957.1 (793, 989, 1096) | 955.5 (794, 929, 1096) |
| Median household income | 55081.0 (47548, 54375, 60178) | 54500.5 (44905, 54002, 60071) | 54820.1 (47094, 54002, 60071) |
| State minimum wage |  |  |  |
| Higher than federal minimum wage | 27 (24.8) | 21 (19.3) | 48 (44.0) |
| Lower than federal minimum wage | 33 (30.3) | 28 (25.7) | 61 (56.0) |
| State refundable EITC rate |  |  |  |
| Yes | 21 (19.3) | 21 (19.3) | 42 (38.5) |
| No | 39 (35.8) | 28 (25.7) | 67 (61.5) |
| Medicaid expansion |  |  |  |
| Yes | 26 (23.9) | 19 (17.4) | 45 (41.3) |
| No | 34 (31.2) | 30 (27.5) | 64 (58.7) |
|  |  |  |  |

* ADV=adolescent dating violence; RJ=restorative justice; YRBS=youth risk behavior survey; TANF=temporary assistance for needy families; EITC= earned income tax credit. Counts are state-years; unweighted percentages are in parentheses, mean (25^th^, 50^th^, 75^th^ percentiles)

##### Supplemental Table 2 Effect modification of state RJ policies on mental health by ethnoracial identity among ADV girl survivors

|  |  | Depressive Symptoms | | | Suicide Ideation | | | | Suicide Attempts | | | |
| --- | --- | --- | --- | --- | --- | --- | --- | --- | --- | --- | --- | --- |
|  | | ARR | 95% CI | *p*-value | ARR | 95% CI | *p*-value | ARR | | 95% CI | *p*-value |  |
| RJ Policy x Hispanic | | 1.01 | (0.97, 1.05) | 0.612 | 1.05 | (0.98, 1.12) | 0.209 | 1.09 | | (0.97, 1.23) | 0.155 |  |
| RJ Policy x Black | | 1.00 | (0.94, 1.06) | 0.969 | 1.01 | (0.92, 1.11) | 0.878 | 1.23 | | (1.06, 1.44) | 0.008 |  |

* RJ = restorative justice; ADV = adolescent dating violence; ARR = adjusted risk ratio; CI = confidence interval. Analyses are unweighted
